# Use of WHO checklist in interventional breast radiological procedures

**DOI:** 10.1186/bcr3794

**Published:** 2015-11-05

**Authors:** Trupti Kulkarni, Andrew O'Connor

**Affiliations:** 1 University Hospital Aintree, Liverpool, UK; 2 Clatterbridge Breast Unit, Wirral, UK

## Introduction

Increasing awareness of safety in healthcare provision has resulted in incorporation of risk-reducing strategies. The WHO checklist is now increasingly being used by interventional radiology. Is it relevant for the interventional breast radiologist?

## Methods

A questionnaire for assessing awareness and use of the WHO checklist used for surgical procedures (or a modified checklist) was devised. Responses were collected and analysed via Survey Monkey.

## Results

Eighty-one complete responses were received and analysed. In total, 93.83 % were aware of the WHO checklist; 83.95 % worked in departments where this was used by IR; and 46.91 % used the checklist individually or as a department. The list was locally devised in 43.21 %. Of those who did not employ use of the list, 27.16 % had considered its use. A total of 24.69 % had never considered using it. Fifty-four per cent opined it was relevant to a therapeutic vacuum-assisted procedure with various individual procedures having scores ranging from 12 to 47 %. Adherence to CQC standards was cited as the reason for use of the checklist. Naysayers quoted increase in time required and poor work flow as reasons for not using it.

## Conclusion

Breast radiological intervention procedures, although low risk and with low complications, remain health interventions. An adverse event should not be a necessary trigger for change of practise. Opinion on use of additional safeguards such as an intervention checklist, while divided, suggested that a modified checklist is called for in complex procedures involving recall of patient at a different date, multiple radiologists involved and therapeutic procedures under vacuum guidance.

